# Establishment of transgenic pigs overexpressing human *PKD2-D511V* mutant

**DOI:** 10.3389/fgene.2022.1059682

**Published:** 2022-11-14

**Authors:** Yuan Zhang, Saifei Xu, Qiao Jin, Jianing Luo, Ce Gao, Sakthidasan Jayaprakash, Huanan Wang, Lenan Zhuang, Jin He

**Affiliations:** ^1^ College of Animal Sciences, Zhejiang University, Hangzhou, China; ^2^ Department of Biotechnology, Hindustan Institute of Technology and Science, Chennai, India

**Keywords:** ADPKD, disease model, *PKD2*, transgenic pig, kidney

## Abstract

Numerous missense mutations have been reported in autosomal dominant polycystic kidney disease which is one of the most common renal genetic disorders. The underlying mechanism for cystogenesis is still elusive, partly due to the lack of suitable animal models. Currently, we tried to establish a porcine transgenic model overexpressing human *PKD2-D511V* (*hPKD2-D511V*), which is a dominant-negative mutation in the vertebrate *in vitro* models. A total of six cloned pigs were finally obtained using somatic cell nuclear transfer. However, five with functional *hPKD2-D511V* died shortly after birth, leaving only one with the dysfunctional transgenic event to survive. Compared with the WT pigs, the demised transgenic pigs had elevated levels of *hPKD2* expression at the mRNA and protein levels. Additionally, no renal malformation was observed, indicating that *hPKD2-D511V* did not alter normal kidney development. RNA-seq analysis also revealed that several ADPKD-related pathways were disturbed when overexpressing *hPKD2-D511V*. Therefore, our study implies that *hPKD2-D511V* may be lethal due to the dominant-negative effect. Hence, to dissect how *PKD2-D511V* drives renal cystogenesis, it is better to choose *in vitro* or invertebrate models.

## Introduction

As the most common renal inherited disease, autosomal dominant polycystic kidney disease (ADPKD) affects millions of people across the world. Progressively enlarged kidneys with numerous fluid-filled cysts are the hallmark of ADPKD, leading to end-stage renal disease in half of the patients in the sixth decade of life (Torres and Harris, 2009). ADPKD is also a systemic disorder with extrarenal manifestations, such as cysts in the liver, pancreas, and spleen, hypertension, cardiac valve malformation, and intracranial aneurysms (Gabow, 1990). Although many people are afflicted by the disease, the only treatment available besides renal replacement therapy is performed by tolvaptan which targets the vasopressin V2 receptor (Torres et al., 2012). However, tolvaptan was reported causing side effects, e.g., hepatic toxicity and nocturia, and only slow down the disease progression (Torres et al., 2017). Thus, novel therapeutic approaches must be developed to reverse renal function loss.

Extensive studies on ADPKD have been conducted for several decades. However, the precise molecular mechanisms responsible for it are still elusive. Thus far, several genes have been reported to be associated with ADPKD, of which *PKD1* and *PKD2* are the main causative genes (Pignatelli et al., 1992; Kimberling et al., 1993; Mochizuki et al., 1996). *PKD1* and *PKD2* encode multiple-transmembrane proteins, polycystin-1 (PC1) and polycystin-2 (PC2), respectively, which form a heterotetrameric protein complex mediating ion influx in the plasma membrane and primary cilium by sensing extracellular mechanical or chemical stimuli (Harris et al., 1995; Qian et al., 2003). Other than primary cilium, PC2 is abundant in the endoplasmic reticulum (ER) (Newby et al., 2002). Initial work suggested PC2 can mediate the influx of calcium, but contrarily, the recent study shows that the PC2 channel has a lower permeability to calcium than potassium and sodium (Koulen et al., 2002; Liu et al., 2018). A newly published paper by Padhy et al. further demonstrated that PC2 in the ER is a potassium channel, which mediates potassium entering the ER and drives calcium release from the ER (Padhy et al., 2022). Thus, it is essential to study the relationship between channel activity and disease phenotype.

Numerous mutations have been deposited in the mutation database of ADPKD (https://pkdb.mayo.edu/). The *PKD2 c.1532A>T/p.511D>V* (hereafter *PKD2-D511V* for gene and PC2-D511V for protein) missense mutation, which results in the replacement of an aspartic acid with valine in the bottom of S3 of PC2, is one of the most extensively studied mutations (Koulen et al., 2002). The genetic studies showed that this mutation impacts the alternative splicing, leading to aberrantly spliced *PKD2* transcripts (Reynolds et al., 1999; Gonzalez-Paredes et al., 2016). The experiments using electrophysiological methods find the mutation is a dominant-negative mutation, which could abolish the channel activity of PC2 (Ma et al., 2005). *In vivo* zebrafish studies on *PKD2-D511V* transient overexpression resulted in the tail curvature, which is a reflection of zebrafish with renal cysts (Feng et al., 2008). Moreover, morpholino targeting zebrafish *pkd2* could not be rescued by the *PKD2-D511V* (Pavel et al., 2016), demonstrating the dominant-negative effect of the mutation. To better characterize the mutation, *in vivo* genetic modified animal models are needed. Currently, only one fruit fly model with transgenic *Amo-D627V* (equivalent to human *PKD2-D511V*) is available to study the molecular consequence of *PKD2-D511V* mutation. However, no dominant-negative effect of the mutation was noticed in the invertebrate model (Köttgen et al., 2011; Hofherr et al., 2016). Although rodent models have proven critical to study the molecular basis of PKD in the past decade, there is no report on *PKD2-D511V* modified mouse model (Happé and Peters, 2014). These results suggest that it is necessary to establish a vertebrate model with *PKD2-D511V* mutation.

Since, the differences in renal anatomy (kidney size and renal function), and life span, a mouse model may not perfectly mimic human ADPKD. Thus, pig might be an ideal alternative to study the disease. Previously, our group constructed a series of pig models with overexpressed *PKD2* and *MYC*, and *PKD1* monoallelic knockout (He et al., 2013; Ye et al., 2013; He et al., 2015). This study aims to construct a transgenic pig model with the human *PKD2-D511V* gene to mimic the ADPKD genotype. Using somatic cell nuclear transfer (SCNT), we obtained six cloned piglets, of which only one cloned pig survived the perinatal stage. Genetic testing showed that the five demised piglets all have *hPKD2-D511V* transgene, while the survived contained a truncated structure. This study is novel on the *PKD2-D511V* vertebrate model, indicating the mutation might be lethal for vertebrates.

## Materials and method

### Plasmids

The plasmids pCAG-WThPKD2-3 × FLAG-floxP-neo-pH11 (hereafter pCAG-WT-hPKD2) and pCAG-muhPKD2 (c.1532A > T/p.511D > V)-3 × FLAG-floxP-neo-pH11 (hereafter pCAG-MU-hPKD2) were constructed in our previous work (Zhang et al., 2020). The pX330-pH11 plasmid, encoding Cas9 protein and gRNA targeting *pH11* locus, was adapted from a previous report using primer annealing and ligation (Ruan et al., 2015). For zebrafish injection and cell transfection, these plasmids were extracted using the EndoFree Midi Plasmid kit (Tiangen, Beijing, China).

### Plasmids injection of zebrafish embryos

The plasmids were diluted to 100 ng/μl with 10% phenol red before injecting into the yolk of fertilized one-cell stage zebrafish eggs. Mock-injected was performed using an equal volume (1 nL) of phenol red solution. The survived fertilized eggs were counted daily, and the dead embryos were discarded. At 5 dpf, the morphological characteristics of zebrafish seedlings were observed under the microscope. The seedlings with typical characteristics were picked for photography. Three biological replicates were performed. The total number of survived zebrafish with mock-injection, and injection of pCAG-WT-hPKD2 and pCAG-MU-hPKD2 were 179, 251, and 82, respectively.

### Generation of transgenic pigs

A million primary CEMP embryonic fibroblast cells were cultured in DMEM (Gibco, Thermo Fisher, Shanghai, China) with 10% fetal bovine serum (Gibco, Thermo Fisher, Australia) at 37°C in a humidified incubator. On the day of transfection, the fibroblasts were digested and mixed with the 3 μg pX330-pH11 and 3 μg pCAG-MU-hPKD2. The transfection was performed using the Lonza Nucleofector (V4XP-3032, Lonza, Germany) with the CA-137 program. Then, the transfected cells were recovered for 24 h in a 6-well-plate before seeding in the 96-well plates at a density of 200 cells/well. 500 ng/μl Geneticin (Thermo fisher, Shanghai, China) was added to each well, and positively selected cells were subcultured to the 24-well-plates after 7–10 days. After that, the cells were split into two aliquots, which were then subjected to PCR identification and cryopreservation. The primers for the identification are detailed in the Supplementary Table S1. Somatic cell nuclear transfer was conducted using the pooled positive cells according to the previously published method (Supplementary Data S1) (Wei et al., 2008). A total of 7 surrogate sows were chosen and received an average of 300 reconstructed embryos. After 121–123 days, the cloned piglets were delivered naturally.

### Genotyping of cloned piglets

Genotyping of cloned pigs was carried out using the same primers to identify positive transgenic cells, except that all the PCR amplified fragments were subjected to sanger sequencing.

### Quantitative real-time polymerase chain reaction and western blotting

Total RNA was isolated from the skin and kidney tissues using the TRIzol Reagent (Tsingke, Beijing, China) according to the manufacturer’s instructions. The RNA was reverse-transcribed (Vazyme, Nanjing, China) following the manufacturer’s instructions, using the HiScript III Reverse Transcriptase (Vazyme, Nanjing, China) to perform the quantitative real-time polymerase chain reactions (qRT-PCR). The primers used in our study were listed in the Supplementary Table S1.

Membrane protein was extracted from the kidney tissues by the membrane and cytosol protein extraction kit (Beyotime, Shanghai, China) for western blotting. Membrane protein was subjected to 8% SDS-polyacrylamide gel electrophoresis. The separated proteins were transferred to PVDF membranes. The membranes were blocked with 5% skimmed milk at 37°C for 2 h, and incubated overnight with diluted primary antibodies against Polycystin-2 (D-3) (PC-2) (1:1000, E20, Santa Cruz Biotechnology, Dallas, TX, United States), DYKDDDDK Tag (FLAG) (1:1000, M2, Sigma-Aldrich, Shanghai, China), followed by goat anti-rabbit IgG (H + L)-HRP (1:5000, BIOKER, Hangzhou, China) and goat anti-mouse IgG (H + L)-HRP (1:5000, BIOKER, Hangzhou, China). An image vertical overflow drain (VCD) gel imaging system was used to determine the optical density (OD) of the protein bands. Finally, the relative abundance was calculated as the ratio of the OD of each protein to the OD of Ponceau S (Beyotime, Shanghai, China) stain.

### Histological examination of cloned piglets

The sample of kidney tissues was fixed with 4% paraformaldehyde, dehydrated, and immersed in transparent wax. Next, the sections were sliced from the prepared paraffin blocks. Then, these sections were stained with hematoxylin and eosin. Antibodies for PC-2 (1:100, E20, Santa Cruz Biotechnology, Dallas, TX, United States) and FLAG (1:100, M2, Sigma-Aldrich, Shanghai, China) were used for immunohistochemistry.

### RNA sequencing

RNA-seq experiments were carried out by Novogene (Beijing, China) using RNA extracted from the porcine kidneys. Following the manufacturer’s recommendations, sequencing libraries were built using the NEBNext UltraTM RNA Library Prep Kit for Illumina (NEB, United States), and index codes were added to attribute sequences to each sample. According to the manufacturer’s instructions, TruSeq PE Cluster Kit v3-cBot-HS (Illumina, United States) was used to cluster the index-coded samples on a cBot Cluster Generation System. The library preparations were sequenced on an Illumina Novaseq 6000 platform, and 150 bp paired-end reads were generated. Next, the cDNA sequence of human *PKD2* was inserted into the *Sus scrofa* reference genome (http://ftp.ensembl.org/pub/release-107/fasta/sus_scrofa/cdna/) before indexing the reference. Salmon was used to align reads to the reference genome and quantify gene expression (Patro et al., 2017). DEGs were screened using the “DESeq2” package (Love et al., 2014) in R software (version 4.1.2) with the cutoff |Log_2_ fold change| > 1 and adjusted *p*-value < 0.05.

The principal component analysis (PCA) was performed by the bioladder cloud platform (https://www.bioladder.cn/) using the whole genome expression profile. The volcano and bubble charts were exported by the “ggplot2″ package (Kolde, 2015; Gómez-Rubio, 2017). The heatmap was exported by the “pheatmap” package (Kolde, 2015).

### Gene ontology functional enrichment analyses

Gene ontology (GO) analyses were performed using the identified DEGs by the Database for Annotation, Visualization and Integrated Discovery (DAVID) with default parameters (https://david.ncifcrf.gov/) (Jiao et al., 2012).

### Statistics

Data were presented as mean ± standard errors of the mean (SEMs). For comparing two groups, either t-test or Mann-Whitney test was used. Pearson’s chi-square test was utilized for comparing category data, and the resulting *p*-values were adjusted with the FDR approach. The type I error was set as 0.05.

## Results and discussion

By accessing the PKD mutation database (https://pkdb.mayo.edu/), there are currently 2600 germline mutation records. However, no mutation hotspot has been identified in both *PKD* genes, indicating that ADPKD mutation is quite family-specific (Rossetti et al., 2001; Gout et al., 2007). Thus, it is unrealistic to establish a representative animal model to study the underline molecular mechanisms. Numerous rodent models have been generated to mimic ADPKD; however, most are null mutation models (Happé and Peters, 2014). Additionally, pig and monkey ADPKD models have been reported using the same strategy by knocking out *PKD1* (He et al., 2015; Tsukiyama et al., 2019; Watanabe et al., 2022). Moreover, approximately 1/3 of the mutations deposited in the database are missense mutations, which might be crucial to understand the disease’s etiology. One of the established missense mutation models is the *PKD1-R3277C* mutation, which has been widely used as the slow progression model of ADPKD (Hopp et al., 2012). Here, we intended to construct a mini-pig model to study the consequence of another missense mutation *PKD2-D511V*.

### Overexpression of *hPKD2-D511V* resulted in tail curvature of zebrafish

**FIGURE 1:**
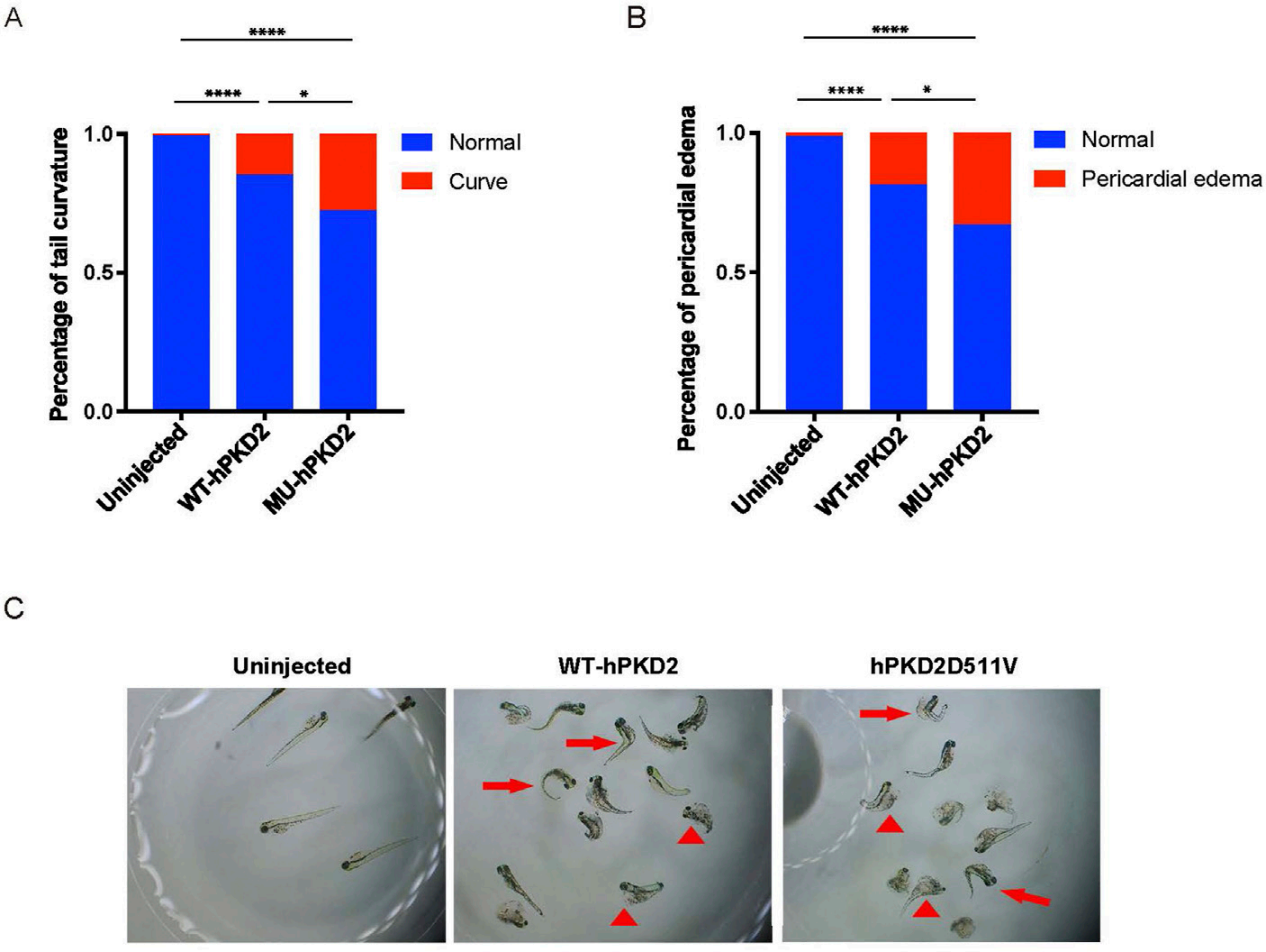
Overexpression of *hPKD2-D511V* resulted in tail curvature of zebrafish. **(A)** Statistical analysis of the percentage of tail curvature zebrafish shows a more severe phenotype in zebrafish injected with the pCAG-MU-hPKD2. **(B)** Statistical analysis of the percentage of pericardial edema zebrafish. **(C)** The morphological observation of zebrafish with pCAG-MU-hPKD2 and pCAG-WT-hPKD2 injection. The arrows indicate tail curvature, and the arrowheads indicate pericardial edema.

Since there is currently no published *PKD2-D511V* vertebrate model to our knowledge. In addition, *in vivo* study demonstrated that *PKD2-D511V* is a dominant-negative mutation related to ion influx. Therefore, generating a porcine model expressing *PKD2-D511V* is risky. The overexpression of the plasmids pCAG-WT-hPKD2 and pCAG-MU-hPKD2 resulted in the enrichment of ADPKD-related pathways in a pig kidney cell line (Zhang et al., 2020). To further prove that *hPKD2-D511V* would be functional *in vivo* and test the consequence of overexpressing the mutant form, the plasmids are injected into the one-cell stage of zebrafish. Unlike other reports, plasmids, not mRNA, were used in our experiment to extend the expression period of *hPKD2*. Similar to previous publications (Feng et al., 2008; Pavel et al., 2016), *hPKD2-D511V* resulted in a more severe tail curvature percentage than WT *hPKD2* injection (Figure 1). Another feature observed is that the *hPKD2-D511V* injected zebrafish had a higher ratio of pericardial edema, implying that heart development was compromised (Figures 1B,C). Zebrafish with induction of acute kidney injury or disturbance of genes related to cilia displayed pericardial edema and ventrally curved tail (Rbaibi et al., 2012; Lee et al., 2015; Wen et al., 2018; Xie et al., 2019). Thus, our zebrafish experiments indicated that the constructed pCAG-MU-hPKD2 vector might be functional *in vivo*.

### Efficient generation of transgenic pigs for *hPKD2-D511V*

**FIGURE 2:**
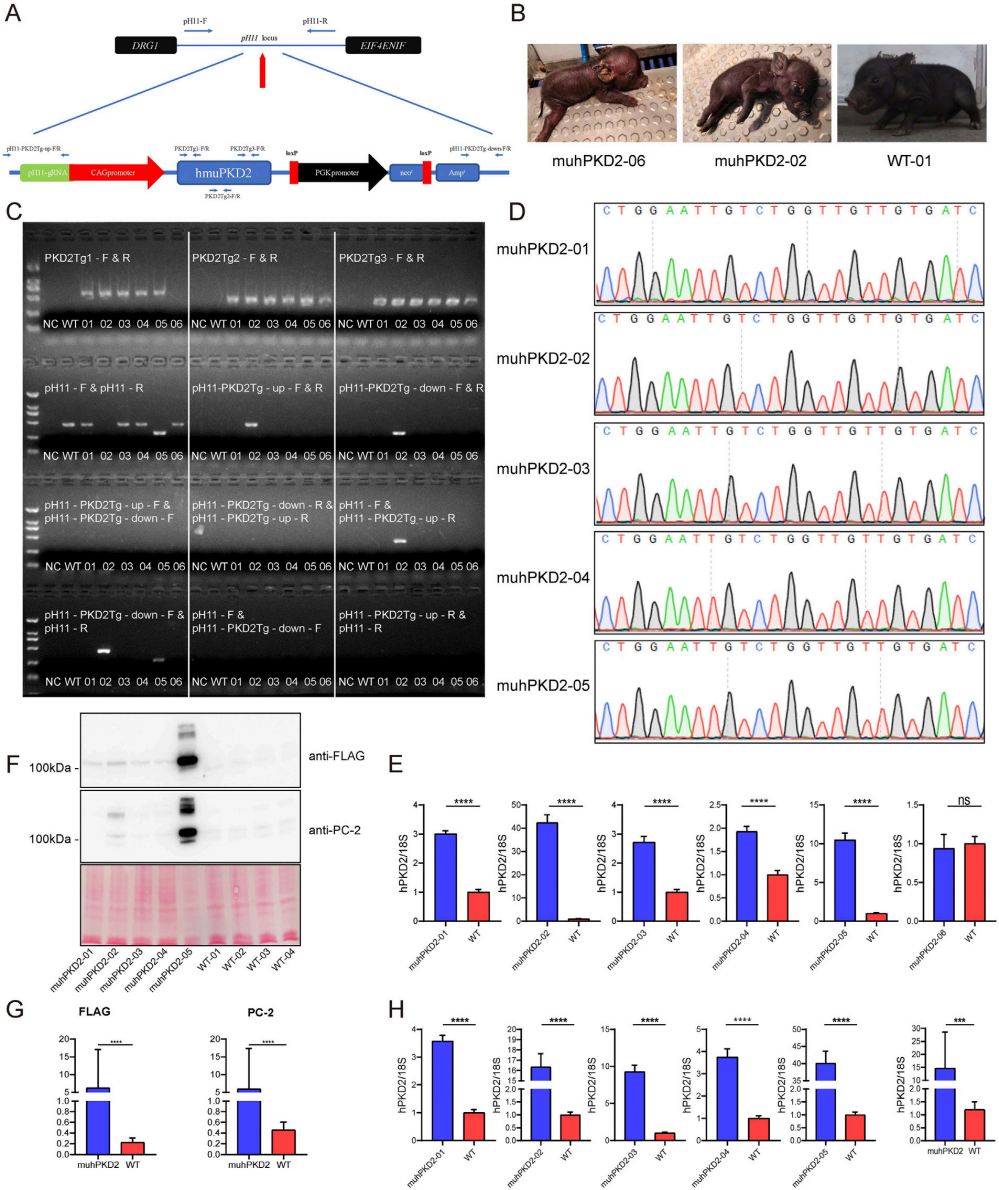
Overexpression of *hPKD2-D511V* resulted in postnatal deaths of the pigs. **(A)** The schematic representation of insertion of the transgene vector to *pH11* safe harbor by the non-homologous end joining mediated targeted insertion. **(B)** Photos of newborn cloned pigs. **(C)** The results of PCR identification. **(D)** Sequencing analyses of cloned pigs using primers PKD2Tg1-F/R. **(E)** The mRNA levels of hPKD2 in the skin tissues of different cloned pigs. **(F,G)** Western blot analysis of FLAG and PC-2 in the cloned pig kidney tissues. Ponceau S staining was used as a loading control in the measurements. **(H)** The mRNA levels of *hPKD2* in the kidney tissues of different cloned pigs. The relative mRNA expression and membrane protein expression levels from the WT group were used as the reference values, and * indicates that there were significant differences between the two groups. Results were expressed as mean ± SEM (*n* = 3).

To precisely analyze the *in vivo* function of *PKD2-D511V*, transgenic pigs were created with *hPKD2-D511V*. To avoid position-effect in transgenic animals, the non-homologous end joining mediated targeted insertion was adapted to insert the transgene vector into the *pH11* safe harbor (Ruan et al., 2015; Zheng et al., 2017). Using this method, Cas9 protein could cut the targets both in the plasmid and genomic DNA. Theoretically, the linearized plasmid could integrate into the *pH11* site of the pig genome. Nevertheless, the linearized plasmid could also integrate into other genomic locations akin to conventional transgenesis (Ju et al., 2015). To preclude the random insertion events, several pairs of primers were designed. Two pairs of upstream and downstream primers could amplify the intended targeted insertion sequence, as shown in Figure 2A. If the vector was inserted reversely, a different combination of these primers could also detect the event (Supplementary Table S1).

The limited dilution method was first utilized for screening the transgenic cells. In this case, if the colonies were formed, all the cells should be derived from the same single cell with an identical genotype. However, due to the status of the available CEMP primary fibroblasts, no cell colonies were formed in our initial screen. Thus, 200 cells/well are seeded to circumvent the problems of obtaining transgenic cells, which would inevitably contain cells with different genotypes. After screening, 16 colonies were secured. PCR identification showed that they all contained transgenic vectors (Supplementary Figure S1). Colonies #3 and #5 were chosen for subsequent SCNT, because they had the intended targeted insertion event, which is presented in Figure 2A. Three batches of embryo transfer were conducted serially; however, only the first batch of embryo transfer resulted in the pregnancy of 2 surrogate sows, which finally delivered 4 and 2 piglets (labeled from MU-hPKD2-01 to -06), respectively. We found that MU-hPKD2-01, 03, 04, and 05 died immediately after birth, while MU-hPKD2-02 died at P1 (Figure 2B). Autopsies showed that the cloned piglets had hepatic congestion, splenomegaly, enlarged pancreas, and lung congestion (Supplementary Figure S2). Only MU-hPKD2-06 survived to date. PCR and sequencing analyses revealed that of the 6 cloned piglets, only MU-hPKD2-02 contained targeted insertion of the transgene in the *pH11* locus, while the others were randomly transgenic events (Figures 2C,D). Interestingly, the survived MU-hPKD2-06 contained a 5′ truncated *hPKD2*, which was validated by qRT-PCR using skin tissues with comparable *hPKD2* levels with the WT piglets (Figure 2E). Thus, the transgenic *hPKD2* might be disrupted in the survived piglet.

Next, the kidneys of the demised pigs were harvested. qRT-PCR and western blotting were then performed to see whether *hPKD2* was elevated in these transgenic pigs. As shown in Figures 2F–H, the transgenic vector could encode the *hPKD2-D511V* in the kidneys at both the mRNA and protein levels.

**FIGURE 3:**
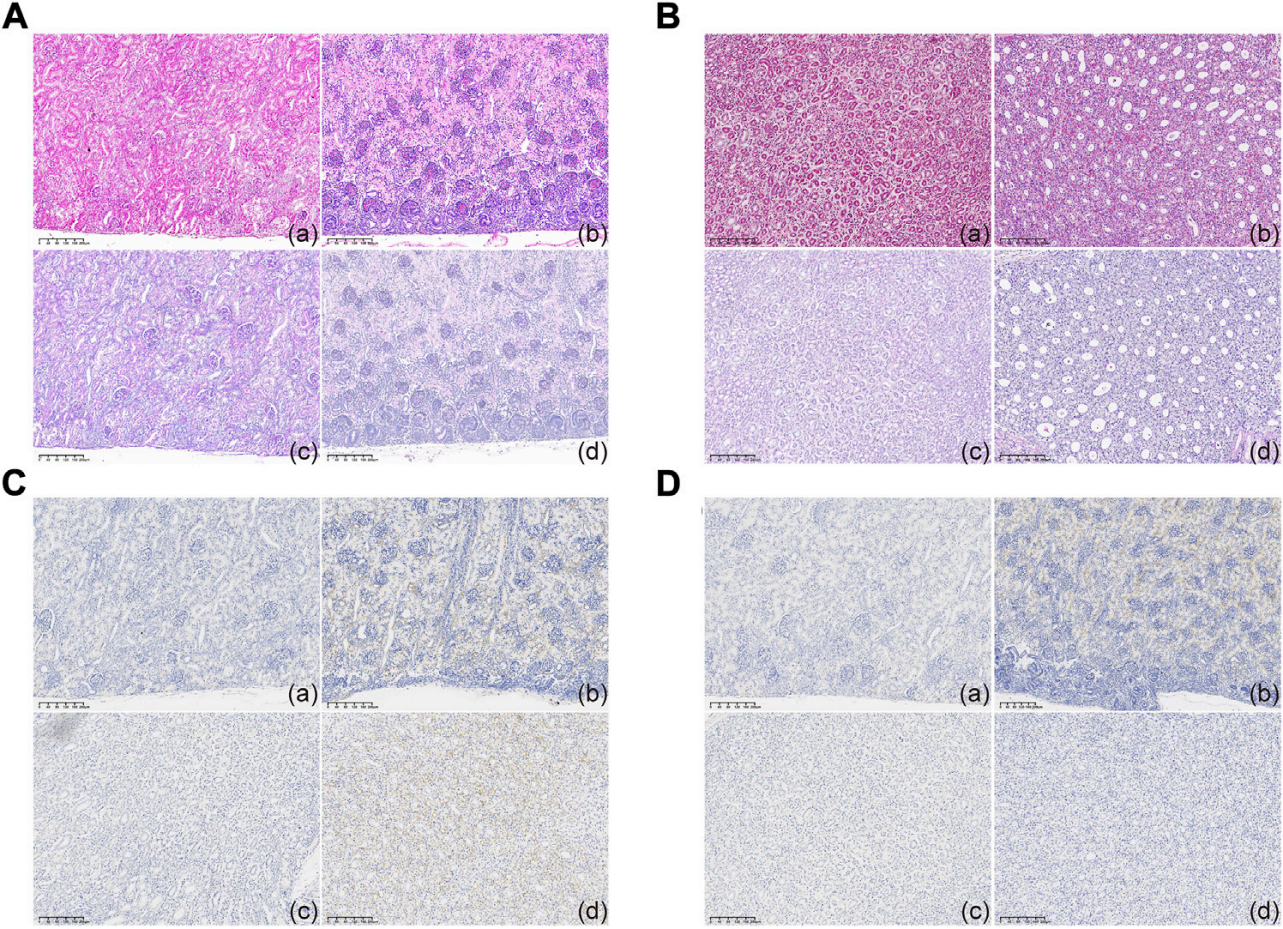
Histological examination of cloned piglets **(A,B)** Optical microscopy observation of the kidney tissues in the MU-hPKD2-02 and WT pigs. **(C,D)** Immunohistochemical characterization of the kidney tissues in the MU-hPKD2-02 and WT pigs. (a and c) MU-hPKD2-02. (b and d) WT pigs. (a,b) renal cortex. (c,d) medulla.

Contrary to the readily detectable *hPKD2* expression, no malformation was noticed in the kidney sections, indicating at least that *hPKD2-D511V* did not affect normal kidney development (Figures 3A,B). Furthermore, immunohistochemical analyses showed increased PC2 in the epithelia of renal tubules when compared to age- and gender-matched WT pigs (Figures 3C,D). These results indicated that the *hPKD2-D511V* overexpression did not elicit the cystic phenotype in newborn piglets. Similar phenomena were also uncovered in our transgenic pigs overexpressing *MYC* and pig *PKD2*, in addition to some rodent models (He et al., 2013; Ye et al., 2013; Cai et al., 2014; Li et al., 2015), making it still divisive whether overexpression model is an explanation to ADPKD (Nagao et al., 2012).

### RNA sequencing reveals the enrichment of ADPKD-related pathways

**FIGURE 4:**
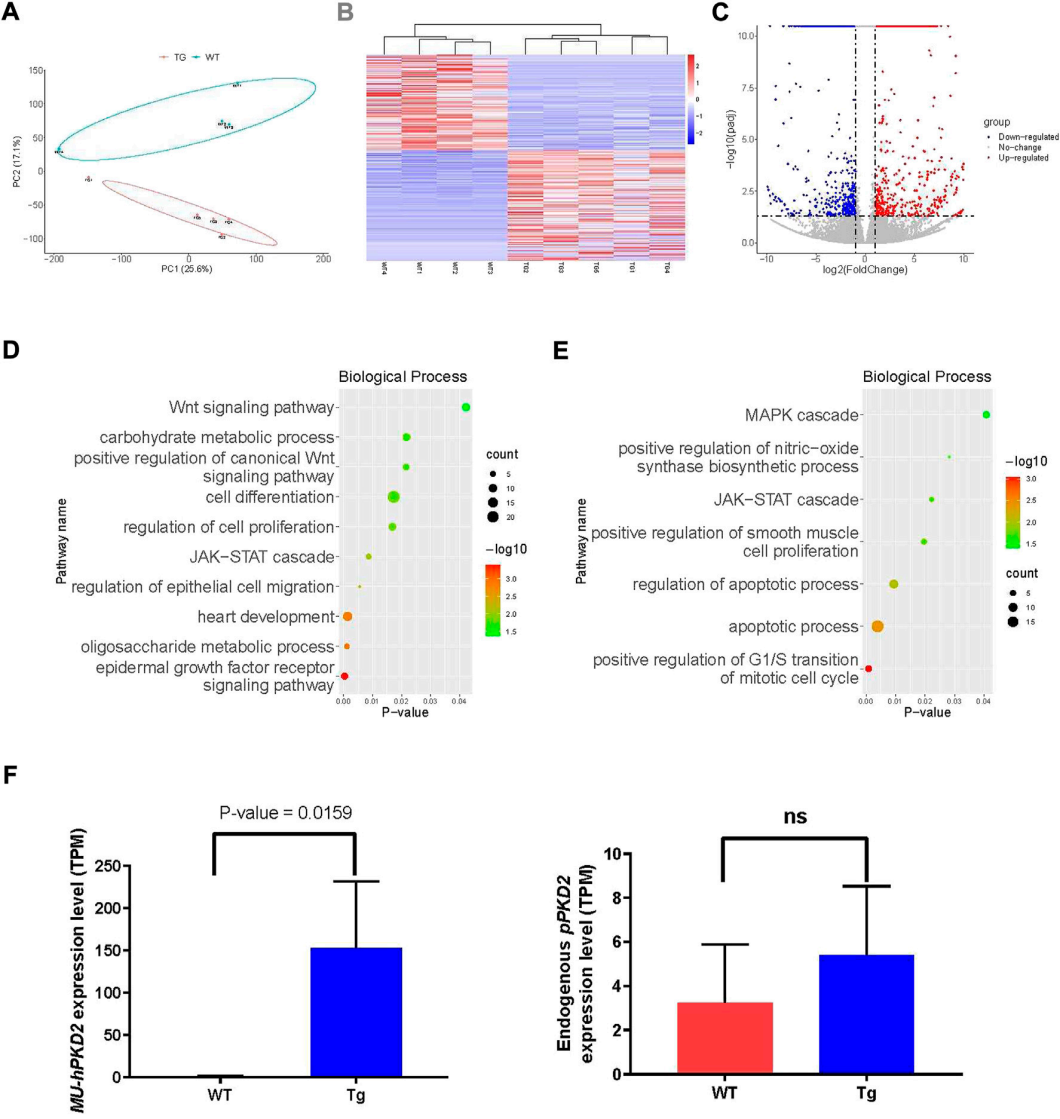
RNA-seq analysis of pig kidneys. **(A)** Principal component analysis (PCA) of merged RNA-seq data indicated the separation of the *hPKD2-D511V* group (MU-hPKD2-01-05) and the WT group (WT-hPKD2-01-04). **(B)** The Heatmap of RNA-seq data with the *hPKD2-D511V* group (MU-hPKD2-01-05) or the WT group based on the Log_2_ intensity. **(C)** Volcano plots showing the adjusted *p*-values and the log_2_ fold change (FC) values of genes in the hPKD2-D511V group (MU-hPKD2-01-05) versus the WT group (WT-hPKD2-01-04). DEGs are indicated by the red or blue dots. **(D,E)** Bubble plots of significant gene ontology (GO) (biological processes) terms enriched in genes from the DEG sets (upregulated DEG for D and downregulated DEG for E) of the two genotypes. **(F)** The TPM of *hPKD2* and *pPKD2* in hPKD2-D511V group (MU-hPKD2-01-05) and WT group.

The kidneys were subjected to RNA-seq due to the lack of renal cystic phenotype to see whether disease-related pathways were disturbed. Four age- and gender-matched WT CEMP piglets were sacrificed, and kidneys were harvested for RNA-seq. The principal component analysis (PCA) showed that overexpression of *hPKD2-D511V* significantly changed the transcriptome compared with the WT group (Figure 4A), which was further confirmed by the heatmap and clustering analysis (Figure 4B). Meanwhile, the volcano plot visualized many differentially expressed genes (DEGs), including 449 upregulated genes and 389 downregulated genes (Figure 4C). GO analysis shows several ADPKD-related biological processes were enriched in our transgenic pigs, e.g., Wnt, MAPK, EGF, JAK-STAT, cell proliferation, apoptosis, and migration (Figures 4D,E) (Bhunia et al., 2002; Wei and Liu, 2002; Ma et al., 2005; Lal et al., 2008). The EGF-mediated ion flux could be activated when overexpression of WT *hPKD*2 in a cell line (Ma et al., 2005). Moreover, this observation could not be replicated when overexpression *hPKD2-D511V*. Our analysis discovered that seven genes were significantly upregulated in *hPKD2-D511V* pig kidneys (Supplementary Table S3), validating the relationship between *PKD2-D511V* and the EGF pathway. One of the pathological hallmarks of ADPKD is massive transcriptomic dysregulation, including EGFR pathway. Some of the major signaling pathways initiated by EGFR activation, and then dependent on the recruitment and binding of specific signaling proteins to the phosphorylated tyrosine residues on the carboxyl termini of EGFR receptor molecules. Further regulate cell function including cell growth, proliferation, migration, differentiation, and apoptosis (He et al., 2013). Besides, the heart development term is also enriched, which might be used to explain the pericardial edema in the zebrafish injected with MU-hPKD2 (Figure 1B). In accordance with qRT-PCR results, the RNA-seq also validated elevated exogenous *hPKD2* transcripts and unaltered endogenous *pPKD2* levels in the transgenic pigs (Figure 4F).

## Conclusion

The current study showed that *hPKD2-D511V* resulted in more tail curvature and pericardial edema in zebrafish experiment, and the *hPKD2-D511V* as a dominant-negative mutation might be lethal in a transgenic pig model. Out of the six cloned piglets, only one survived; moreover, the survived one contained a truncated form of the transgene that would not be expressed at the mRNA level (Figure 2E). Therefore, to study the molecular mechanism of *PKD2-D511V* driving cystogenesis in human patients, other approaches, such as transient expression in zebrafish embryos or the construction of non-invertebrate models, should be considered.

## Data Availability

The datasets presented in this study can be found in online repositories. The names of the repository/repositories and accession number(s) can be found below: NCBI SRA, PRJNA881011.
