# Supplementary figures and images for: Establishment of transgenic pigs overexpressing human PKD2-D511V mutant
Source: Front Genet. 2022 Nov 14;13:1059682. doi: 10.3389/fgene.2022.1059682 (PMC9702356; doi:10.3389/fgene.2022.1059682)

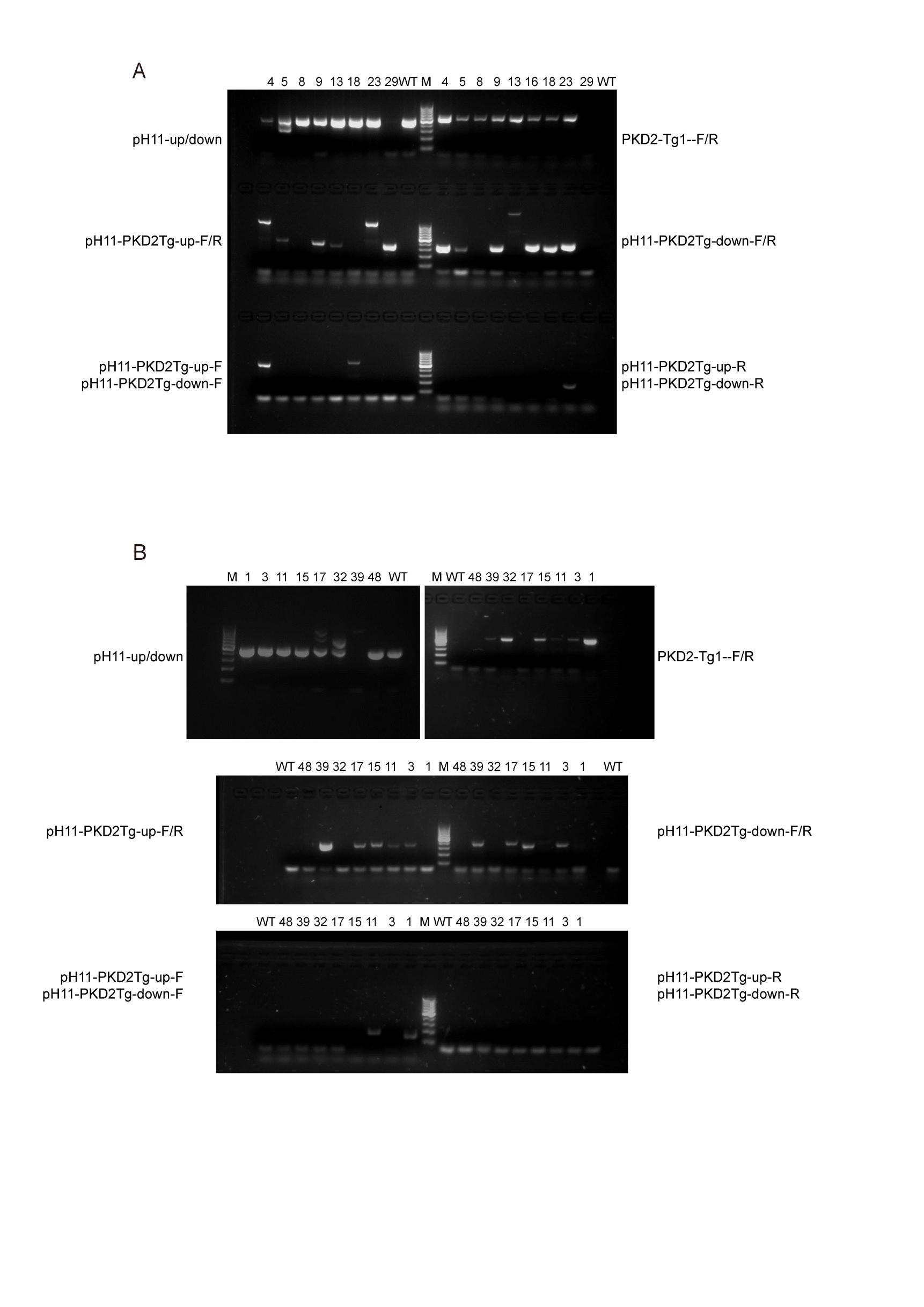

Supplement: Supplementary file 1 [file Image1.JPEG]

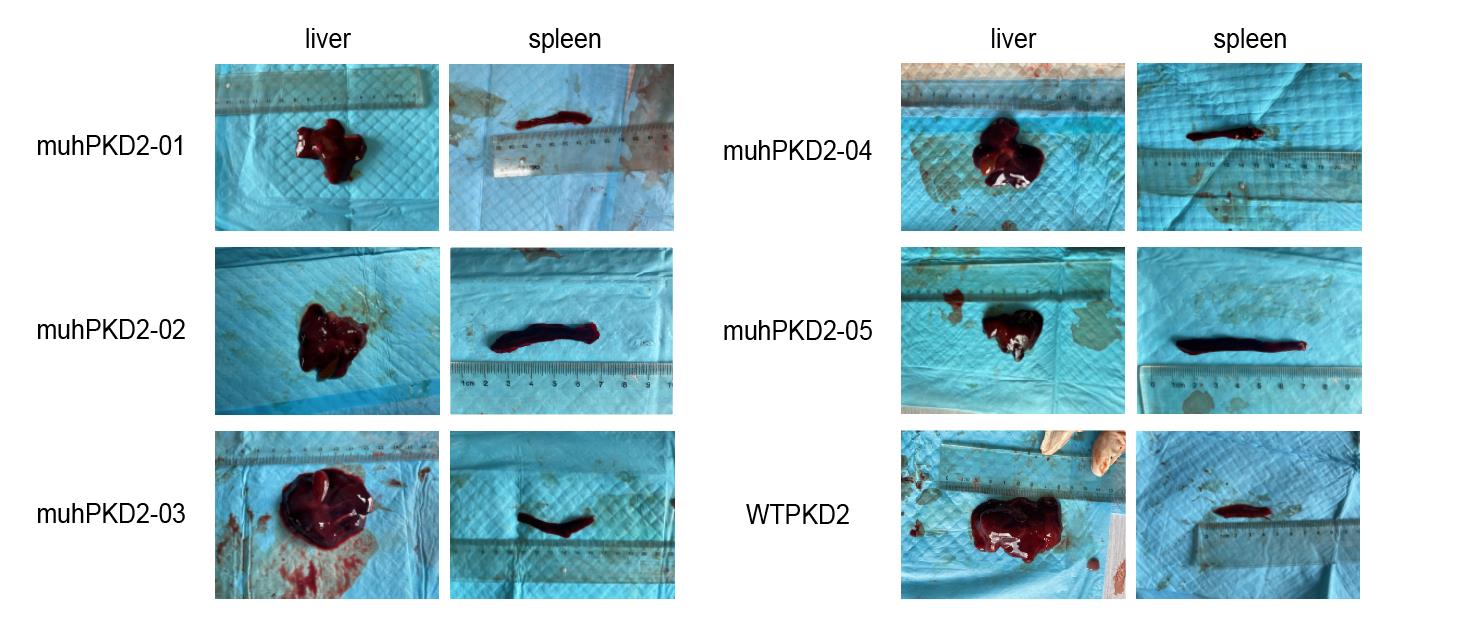

Supplement: Supplementary file 2 [file Image2.JPEG]
