# Supplementary material for: Establishment of transgenic pigs overexpressing human PKD2-D511V mutant
Source: Front Genet. 2022 Nov 14;13:1059682. doi: 10.3389/fgene.2022.1059682 (PMC9702356; doi:10.3389/fgene.2022.1059682)
Supplement: Supplementary file 4 [file DataSheet1.DOCX]

Supplementary Material

# Supplementary Data

## Somatic cell nuclear transfer

Nuclear transfer was conducted by using inverted microscope TE2000U equipped with manipulation system (Eppendorf, Germany). Micromanipulation drops consisted of Hepes buffered NCSU-23 supplemented with 7.5 μg/ml cytochalasin B (CB) covered with mineral oil. Receptor cell selected the oocytes with constant cytoplasm, and removed the first polar body and 10–15% of its surrounding cytoplasm probably containing metaphase plate of oocytes. Donor cells selected the positive cells authenticated by PCR. Then, injected the donor cells into the perivitelline space through the same slit. Two DC pulses of 1.6 kV/cm for 100 μs each at 100 μs interval, delivered by a CUY-21 electroporator (BEX, Japan), induced fusion and activation. After NT, reconstructed couplets were transferred into drops of media covered with mineral oil for 1–2 h recovery until fusion and activation were carried out.

## 1.2 Transfected cells identification and the autopsied of cloned piglets

The transfected cells subjected to PCR identification (Supplementary Figure 1). The primers for the identification are detailed in the supplementary table 1. Somatic cell nuclear transfer was conducted using the pooled positive cells according to the previously published method. A total of 7 surrogate sows were chosen and received an average of 300 reconstructed embryos. After 3 months, the cloned piglets were delivered naturally. Unluckily, MU-hPKD2-01, 03, 04, and 05 died immediately after birth, while MU-hPKD2-02 died at P1 (Figure 2B). Autopsies showed that the cloned piglets had hepatic congestion, splenomegaly, enlarged pancreas, and lung congestion (Supplementary Figure 2).

# Supplementary Figures and Tables

##
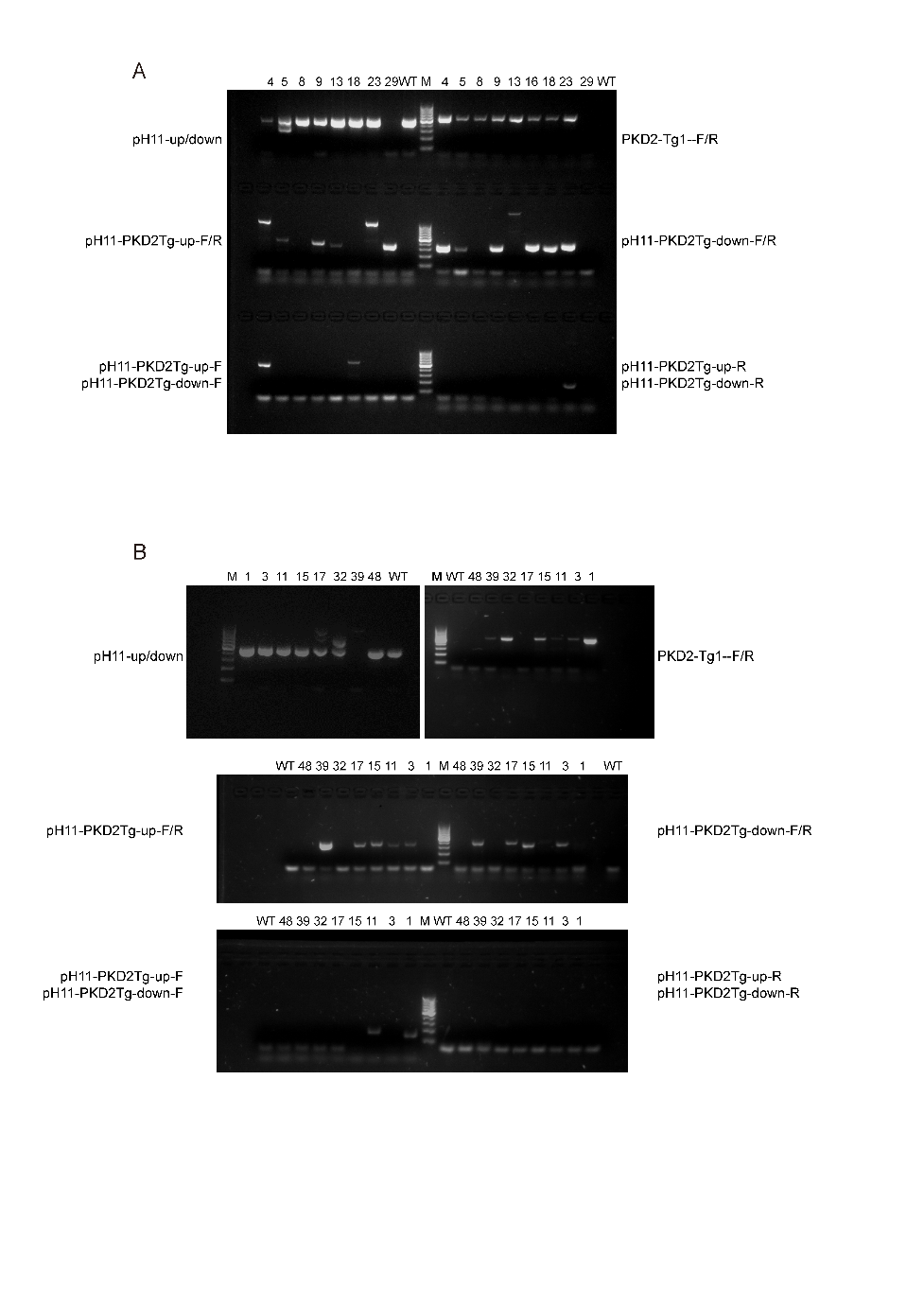
Supplementary Figures

**Supplementary Figure 1.** The results of PCR identification of the transfected cells used the primer shown in Figure 3A.


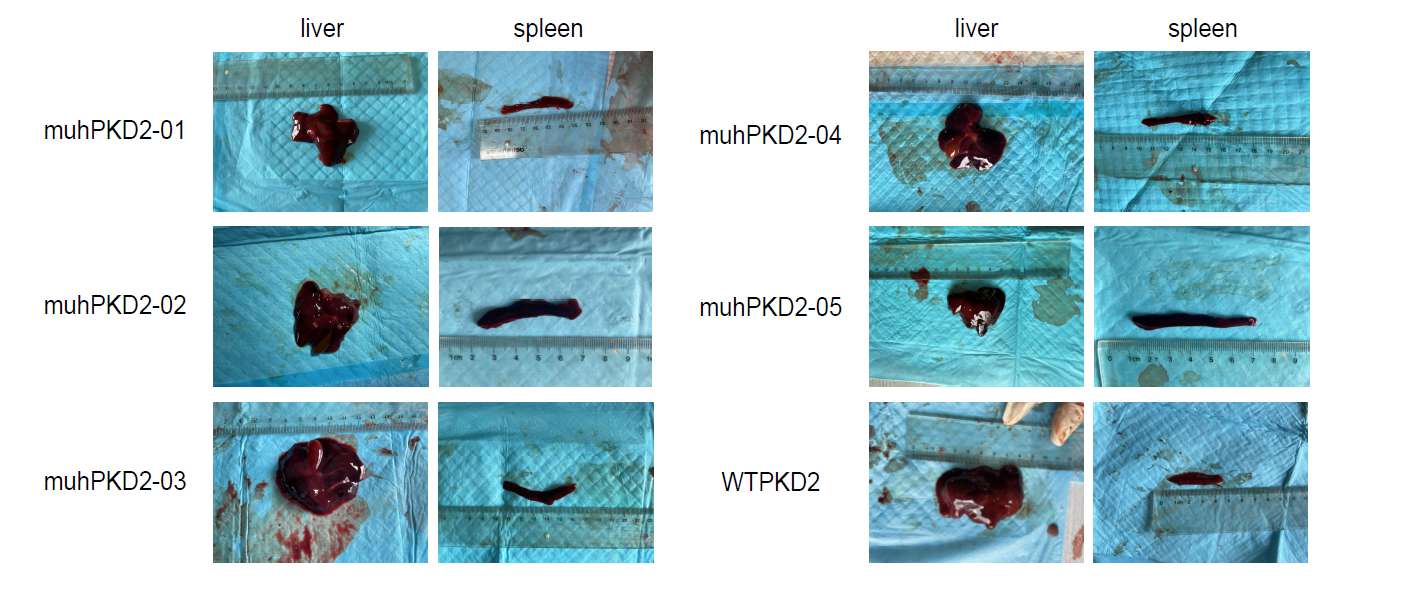


**Supplementary Figure 2.** Autopsies showed that the cloned piglets had hepatic congestion, splenomegaly, enlarged pancreas, and lung congestion.
